# A Loop Region in the N-Terminal Domain of Ebola Virus VP40 Is Important in Viral Assembly, Budding, and Egress

**DOI:** 10.3390/v6103837

**Published:** 2014-10-17

**Authors:** Emmanuel Adu-Gyamfi, Smita P. Soni, Clara S. Jee, Michelle A. Digman, Enrico Gratton, Robert V. Stahelin

**Affiliations:** 1 Department of Chemistry and Biochemistry, the Eck Institute for Global Health, University of Notre Dame, Notre Dame, IN 46556, USA; E-Mails: adugee@gmail.com (E.A.-G.); clarasyjee@gmail.com (C.S.J.); 2 Department of Biochemistry and Molecular Biology, Indiana University School of Medicine-South Bend, South Bend, IN 46617, USA; E-Mail: ssoni@iupui.edu; 3 Department of Biomedical Engineering, University of California, Irvine, CA 92697, USA; E-Mails: mdigman1@gmail.com (M.A.D.); egratton@uci.edu (E.G.); 4 Centre for Bioactive Discovery in Health and Ageing, School of Science and Technology, University of New England, Armidale NSW 2351, Australia

**Keywords:** Ebola virus, filovirus, number and brightness analysis, plasma membrane, viral budding, VP40

## Abstract

Ebola virus (EBOV) causes viral hemorrhagic fever in humans and can have clinical fatality rates of ~60%. The EBOV genome consists of negative sense RNA that encodes seven proteins including viral protein 40 (VP40). VP40 is the major Ebola virus matrix protein and regulates assembly and egress of infectious Ebola virus particles. It is well established that VP40 assembles on the inner leaflet of the plasma membrane of human cells to regulate viral budding where VP40 can produce virus like particles (VLPs) without other Ebola virus proteins present. The mechanistic details, however, of VP40 lipid-interactions and protein-protein interactions that are important for viral release remain to be elucidated. Here, we mutated a loop region in the N-terminal domain of VP40 (Lys^127^, Thr^129^, and Asn^130^) and find that mutations (K127A, T129A, and N130A) in this loop region reduce plasma membrane localization of VP40. Additionally, using total internal reflection fluorescence microscopy and number and brightness analysis we demonstrate these mutations greatly reduce VP40 oligomerization. Lastly, VLP assays demonstrate these mutations significantly reduce VLP release from cells. Taken together, these studies identify an important loop region in VP40 that may be essential to viral egress.

## 1. Introduction

Ebola virus (EBOV) [1] and Marburg virus (MARV) [2] are viruses from the *Filoviridae* family, which are some of the most virulent pathogens that infect humans. These infections cause severe hemorrhagic fevers with fatality rates of ~60% and, to date, there have been no vaccines or therapeutic treatments approved by the The Food and Drug Administration (FDA). EBOV is filamentous in shape and the genome consists of single stranded negative sense RNA encoding seven proteins. The glycoprotein (GP) is exposed on the surface of the viral envelope and is responsible for entry of the virions [3] through an interaction with Niemann-Pick C1 receptor in the host cell [4,5]. The matrix protein viral protein 40 (VP40), which associates with the viral lipid coat, is important for EBOV budding as well as virus structure and stability [6,7]. The nucleocapsid (NC), made of nucleoprotein (NP), VP24, VP30, VP35 and L protein, is crucial for viral transcription and replication [8]. In the absence of VP40, the NC is inefficiently transported to the plasma membrane (PM) and virus particles do not form [9].

**Figure 1:**
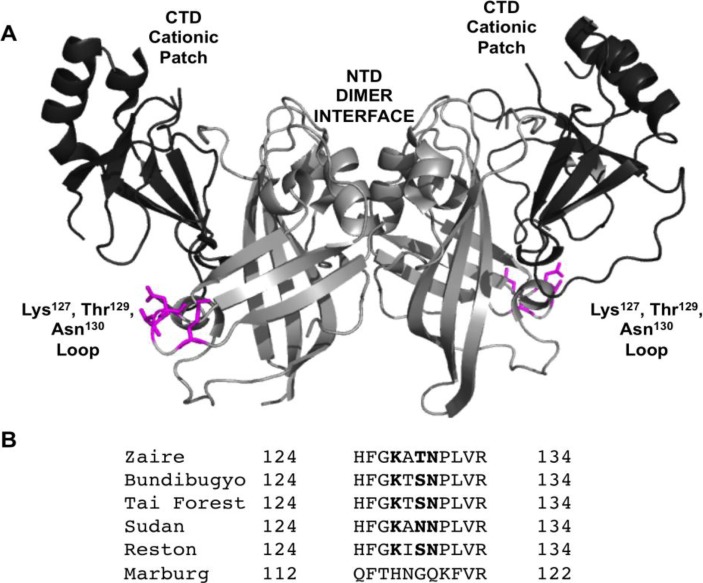
VP40 harbors a N-terminal and C-terminal domain. (**A**) VP40 (PDB ID: 4LDB) is a dimer mediated by a N-terminal domain (NTD) interface. The C-terminal domain (CTD) mediates membrane binding through a cationic patch and also creates an interface for VP40 oligomerization [13]. The NTD is shown in light gray and the CTD in dark gray. Residues found to be important in this study (Lys^127^, Thr^129^, and Asn^130^) for VP40 PM localization, oligomerization, and budding are shown in magenta. (**B**) VP40 sequence alignment from ebolaviruses and Marburg virus for the N-terminal loop region studied herein. Residues found to reduce PM localization, oligomerization, and VLP formation for EBOV VP40 are shown in bold.

VP40, in the absence of the other six EBOV proteins, has been shown to harbor the ability to form virus like particles (VLPs) when expressed in human cells [10]. The VLPs that are released in response to VP40 expression resemble authentic Ebola virions [6,7,11,12]. Thus, VP40 has been used to study protein-protein and lipid-protein interactions as it serves as a representative model of budding and can be used in laboratory environments without BSL-4 facilities. VP40 harbors a N-terminal domain (NTD) that regulates dimerization [13] and a C-terminal domain (CTD) that has been shown to mediate membrane binding [7,13,14,15,16,17,18] and oligomerization [13] (See Figure 1). Inhibition of VP40 oligomerization is an effective means of halting budding. Elucidation of VP40’s role in the VLP assembly and release is essential to identifying sites that may be targeted to inhibit the replication and spread of the virus [19]. Interactions of VP40 with microtubules [20], actin [21,22], and IQGAP1 [23] have been observed and in the case of actin may guide VP40 movement and assembly [21]. Additionally, VP40 has been shown to interact with the COPII protein Sec24C [24], as well as Tsg101 [25], which is part of the ESCRT-I complex.

Specific lipid interactions are not yet known, but phosphatidylserine, an anionic lipid enriched in the PM inner leaflet, is important for C-terminal domain membrane interactions [14,15,17,18]. Specifically, a cationic patch in the C-terminal domain is thought to mediate association with anionic membranes [13] while a hydrophobic loop mediates penetration of the domain into the hydrocarbon core of the bilayer [14,17]. VP40 has also been shown to play an important role in viral transcription through formation of a RNA binding octameric ring [13,26], but the octameric ring has not been observed in infectious virions or VLPs. While a number of regions of VP40 that are key determinants of trafficking or release have been identified, much less mechanistic information is available on the molecular basis of VP40 interactions with human proteins or cellular membrane lipids.

In this study we generated several mutations of the VP40 N-terminal domain to investigate regions of VP40 that may be essential to VLP formation. Using a combination of cellular imaging, number and brightness analysis (N&B), and VLP release assays we find that a loop region in the N-terminal domain is important for VP40 PM localization, VP40 oligomerization, and VLP release. In contrast, mutations of cationic residues in the N-terminal domain outside of this loop region (K86A or K90A) did not appreciably alter PM localization of VP40 or VLP release. Thus, this N-terminal domain loop region may play an important role in VP40-protein or VP40-membrane interactions with the host cell.

## 2. Experimental Section

### 2.1. Materials

Restriction endonucleases and enzymes for molecular biology were obtained from New England Biolabs (Beverly, MA, USA). The QuikChange site-directed mutagenesis kit was from Agilent Technologies, Santa Clara, CA, USA. Nunc Lab-Tek I Chambered Cover Glasses 8-well and bicinchoninic acid (BCA) protein assay kit were from Thermo Fisher Scientific (Waltham, MA, USA). Lipofectamine 2000 and Lipofectamine LTX were from Invitrogen (Carlsbad, CA, USA).

### 2.2. Molecular Biology and Protein Expression

VP40 mutants were generated in pcDNA3.1 using a QuikChange site-directed mutagenesis kit and then subcloned into the pGEX-4T-1 vector using the EcoRI and XhoI restriction sites. The introduced mutations were confirmed by automated DNA sequencing using the primer 5’ CCG GAA TTC GCC ATG AGG CGG GTT ATA 3’. VP40 and respective mutations were purified to homogeneity as previously described [14,17]. Circular dichroism of WT VP40 and mutations was performed as previously described [17].

### 2.3. Cell Imaging

HEK293 cells were cultured and maintained at 37 °C in a 5% CO_2_ humidified incubator supplemented with DMEM (low glucose) containing 10% FBS and 1% Pen/Strep. After trypsinization, cells were transferred from a T-25 tissue culture flask to an 8-well plate used for imaging. Cells were then grown to 50%–80% confluency and transfected with 1 µg DNA/dish using lipofectamine 2000 according to the manufacturer’s protocol. CHO-K1 cells were cultured and maintained at 37 °C in a 5% CO_2_ humidified incubator supplemented with DMEM/F12 (low glucose) containing 10% FBS and 1% Pen/Strep. After trypsinization, cells were transferred from a T-25 tissue culture flask to an 8-well plate used for imaging. Cells were then grown to 50%–80% confluency and transfected with 1 µg DNA/dish using lipofectamine LTX according to the manufacturer’s protocol. Cells were imaged using a Zeiss LSM 710 confocal microscope using a Plan Apochromat 63× 1.4 NA oil objective. The 488 nm line of the Ar ion laser was used for excitation of EGFP. The laser power was maintained at 1% throughout the experiment with the emission collected through a 493–556 nm filter.

### 2.4. Total Internal Reflection Fluorescence Microscopy Imaging

Total Internal Reflection Fluorescence (TIRF) microscopy imaging was performed using a homebuilt TIRF imaging system (model No. IX81 microscope (Olympus, Melville, NY, USA) as described previously [27]. For N&B analysis, 512 frames were analyzed per image series. HEK293T cells expressing monomeric EGFP used as a brightness standard were imaged under the same conditions as EGFP-VP40 and respective mutations. The brightness of the EGFP was used as the brightness of the monomer (Figure 4). The selection window for analysis in the brightness *versus* intensity plot was based on the average brightness of a monomer (0.104), which allowed for selection of oligomeric size based upon addition of each monomer to yield, dimer, trimer, *etc.* Thus, the selection window for each species is based upon the average brightness, which will yield an average population of each species in the respective area of analysis.

N&B analysis is based upon moment analysis and allows for measurement of the average number of molecules as well as brightness in each pixel of a fluorescent microscopy image [27]. Here, the average brightness of a particle is determined from the ratio (of variance to intensity) at each pixel. Fluctuating particles can be determined by dividing the average intensity by the brightness at each pixel. In live cells, intensity may be attributed to autofluorescence, scattering, bright immobile particles, or fast moving particles that are dim. To control for these parameters, the N&B analysis accounts for these limitations by calculating the total variance, which also incorporates the detector noise. For particles fluctuating in the focal volume, the variance is proportional to the particle brightness; however, the variance of the immobile particles, scattering, autofluorescence, and detector noise is proportional to the intensity of these components. Thus, only fluorescent fluctuations that are dependent upon the mobile particles (square of the brightness) have a ratio of the variance to intensity >1. Brightness maps then allow for pixel resolution of the clustering of fluorescently labeled proteins.

For the electron multiplying CCD camera the following equation was used to compute the number and brightness.

*N* = (< *I* > - offset)^2^/(*σ*^2^ − *σ*_0_^2^) (1)

B = (σ^2^ − σ_0_^2^)/(< *I* > - offset) (2)

where N and B are the apparent number and the brightness of the molecule, < *I* > is the average intensity, σ^2^ is the variance and *offset* and σ_0_^2^ are the intensity and noise variance due to the camera. With these parameters properly calibrated using EGFP, we obtained the distribution of the brightness of each individual pixel in the image of the cell under investigation.

### 2.5. VLP Assays

To determine if VLPs were efficiently released for different mutations compared to WT, EGFP-VP40, EGFP-VP40-K86A, EGFP-VP40-K90A, EGFP-VP40-K127A, EGFP-VP40-T129A, EGFP-VP40-N130A or EGFP were expressed in CHO-K1 cells for 48 hours. VLPs were then isolated from cellular media as previously described [14,17,21]. Cell lysates and VLP samples were mixed with SDS loading buffer and were boiled before loading on an 8% SDS-PAGE gel. The gel was then transferred to a nitrocellulose membrane (Bio-Rad) that was later incubated with primary rabbit polyclonal anti-EGFP antibody (Thermo Scientific) (1:1000 dilutions) or rabbit polyclonal anti-GAPDH (Santa-Cruz Biotechnology) followed by goat anti-Rabbit HRP (Bio-Rad) conjugate as secondary antibody according to the manufacturers protocol (Thermo Scientific). Blots were exposed using ImageQuant LAS 4000 (GE Healthcare). This method allows a comparison of VP40 in VLPs *versus* cell lysate using GAPDH as a loading control. Experiments were performed in triplicate.

## 3. Results and Discussion

### 3.1. VP40 Protein-Protein and Lipid-Protein Interactions

A number of regions and amino acids in VP40 have been found to be important for VP40 trafficking [24], PM localization [25,28,29], membrane binding [7,13,14,17], oligomerization [13], and protein-protein interactions. For instance, VP40 has a N-terminal region containing two overlapping late-domains (L-domains), PTAP and PPxY that interact with Tsg101 [25] and Nedd4 [30], respectively. The C-terminal domain, which has been shown to mediate the association of VP40 with membranes also can insert into the membrane bilayer with an exposed hydrophobic patch that favors membrane penetration to the inner leaflet of the PM [14,17]. Despite these discoveries little mechanistic information is available on the molecular basis of VP40-protein or VP40-lipid interactions and a number of VP40 interactions with proteins or lipids likely remain unknown.

**Figure 2:**
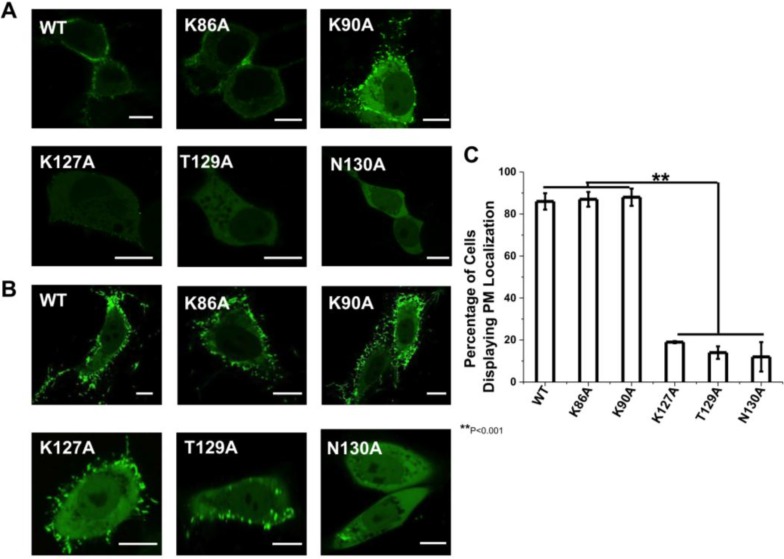
Cellular localization of VP40 and mutations in HEK293 and CHOK-1 cells. (**A**) HEK293 cells were grown in an 8-well plate and transfected with WT VP40, K86A, K90A, K127A, T129A, and N130A DNA containing an EGFP fusion tag. Cells were imaged after 20 h using a Zeiss LSM 710 confocal microscope with a 63× 1.4 numerical aperture oil objective. Scale bar = 10 µm. (**B**) CHOK-1 cells were grown in an 8-well plate and transfected with WT VP40, K86A, K90A, K127A, T129A, and N130A DNA containing an EGFP fusion tag. Cells were imaged after 16 h using a Zeiss LSM 710 confocal microscope with a 63× 1.4 numerical aperture oil objective. Scale bar = 10 µm. (**C**) A histogram was plotted to demonstrate the percentage of HEK293 cells that displayed detectable PM localization for WT VP40 and mutations. Experiments were repeated in triplicate using at least 100 cells in each experiment to determine the S.D. as shown. One-way ANOVA analysis was used to calculate the standard error of the mean and *p*-value. ** *p* < 0.001.

**Figure 3:**
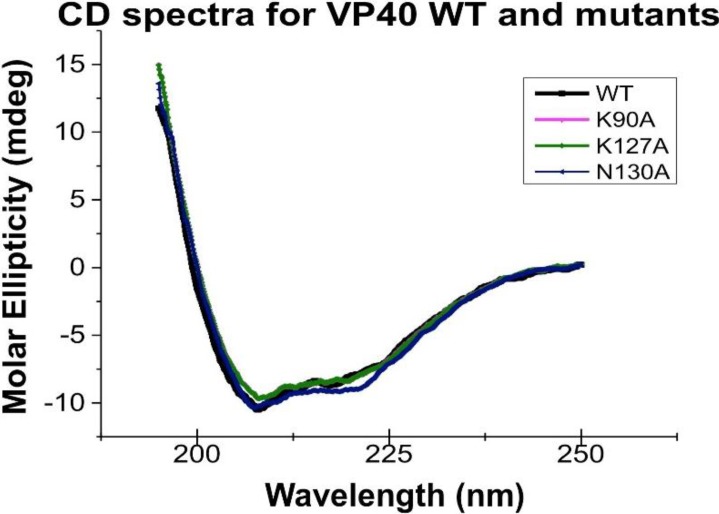
Circular dichroism spectra of VP40 and mutants employed in this study. The spectra were taken on a JASCO 815 CD spectrometer scanned from 195–250 nm in a 1 mm quartz spectrophotometer cell. Each measurement was performed with 5 mg of indicated protein and was performed in triplicate to yield the mean representative scans. Molar ellipticity was defined according to the JASCO software [31] and was subtracted from a control buffer scan.

To further screen for regions of VP40 that are important for PM localization and viral egress we randomly introduced mutations into the VP40 N-terminal domain to screen for changes in PM localization. To this end, three mutations showed distinct changes in the initial screen and included K127A, T129A, and N130A, which were predominately localized to the cytoplasm of HEK293 and CHOK-1 cells (Figure 2). In contrast, other mutations that reduced positive charge in the N-terminal domain (K86A and K90A) did not show appreciable changes in PM localization in the initial screen and were used as controls. To determine if any major structural changes occurred for these mutations we performed circular dichroism (CD) spectroscopy on WT VP40 and the respective mutations. As shown in Figure 3, mutations and WT had overlapping spectra indicating that no major structural changes occurred for VP40. Thus, we decided to more intensely investigate these five mutations in the PM localization, oligomerization and VLP release of VP40.

### 3.2. VP40 Cellular Localization

The cellular localization of VP40 and respective mutations were analyzed in HEK293 and CHOK-1 cells. To analyze the effect of the mutant’s in cells, we transfected HEK293 cells with plasmids encoding WT VP40, K86A, K89A, K127A, T129A and N130A, all of which harbor an N-terminal EGFP tag. The cells were imaged using confocal microscopy 20 h post-transfection in HEK293 cells and 16 h post-transfection in CHOK-1 cells. As shown in Figure 2A, WT EGFP-VP40 is predominantly localized to the PM with strong visual evidence of VLP extensions stemming from the PM. In contrast, T129A and N130A display a diffuse cytosolic localization and a significant reduction in PM localization (Figure 2A). K127A also significantly reduced PM localization and evidence of VLP egress but to a lesser extent than T129A and N130A. For these three mutations, ~10%–20% of cells exhibited visually detectable PM localization (Figure 2C). Moreover, when PM localization of K127A, T129A, or N130A was detectable, the amount of VP40 localized to the PM was much lower than WT EGFP-VP40 expressing cells (see more details below). K86A and K90A displayed similar PM localization and formation of filamentous extensions in HEK293 cells and did not display a statistically significant reduction in PM localization (Figure 2C). To further quantify the PM localization of VP40 and respective mutations, PM intensity determination was performed using the Zeiss software [32] to determine the relative intensity of the EGFP signal in at least five regions of interest (ROI, same area of analysis) in HEK293 cells. The cytoplasmic intensity of EGFP was then measured in 5 distinct ROIs of the same area. These average signals were used for assessing the ratio of PM VP40 EGFP intensity/(total VP40 EGFP intensity (cytoplasmic + membrane)) to provide a quantitative estimate of PM localization. Seven different cells for each construct, which were repeated in triplicate on different days, were used to determine the relative PM localization of EGFP-VP40 and respective mutations based upon EGFP intensity. The analysis revealed the average PM intensity to be 87% ± 6% for WT, 86% ± 5% for K86A, 82% ± 8% for K90A, 13% ± 3% for K127A, 7% ± 3% for T129A and 8% ± 2% for N130A, yielding similar results to the number of cells displaying PM localization that was determined by visual inspection (Figure 2C).

Similarly, WT, K86A, and K90A had extensive PM localization in CHOK-1 cells (Figure 2B) while K127A and T129A had a significant reduction in PM localization harboring some signal on the PM and a small amount of filamentous protrusions extending from the PM. N130A, however, displayed little detectable PM localization in CHOK-1 cells. Quantification of PM localization in CHOK-1 cells was only performed by counting cells that had visually detectable PM localization.

### 3.3. VP40 Oligomerization

To investigate if the N-terminal domain mutations altered VP40 oligomerization in HEK293 cells we used TIRF microscopy and N&B analysis [14,27] to study the oligomerization state of VP40 and mutants at the PM. VP40 oligomerizes on the inner leaflet of the PM into hexamers and larger oligomers that are enriched in PM protrusion sites [14,27]. TIRF is well suited for this approach as it excites fluorescent molecules on or near the PM while N&B analysis allows for measurement of the average number of molecules as well as brightness in each pixel of a fluorescent image. Thus, this method is well suited to resolve the spatial distribution of VP40 and VP40 mutant oligomers in live cells [27]. The calibration with monomeric EGFP allows for the selection of oligomers based upon the brightness of the monomer. Thus, selection windows in the brightness *versus* intensity plots can be used to select the average brightness of each species of interest (monomer, dimer, hexamer, *etc.*) to visualize the cellular localization of each species. The average brightness of a monomer was 1.104 in the brightness *versus* intensity plot meaning the brightness of a dimer was 1.208 and that of a trimer was 1.312. Thus, we chose to analyze monomers and dimers together as gleaning the accurate separation in the brightness *versus* intensity plot (difference of 0.104 units of brightness) is difficult to interpret accurately. Similarly, we have chosen to analyze trimers and dimers together (difference of 0.1 units of brightness) and molecules that are hexameric and larger together. This provides a means of analyzing VP40 assembly into hexamers that may concatenate into larger filaments at the PM as previously reported [13,14,27]. Additionally, these larger assemblies are needed to induce PM structural changes and VLP egress. Specifically, we compare the formation of VP40 oligomers that are hexameric and larger to that of VP40 monomers and dimers. This allows for assessment of a statistically significant reduction in oligmerization of VP40.

**Figure 4:**
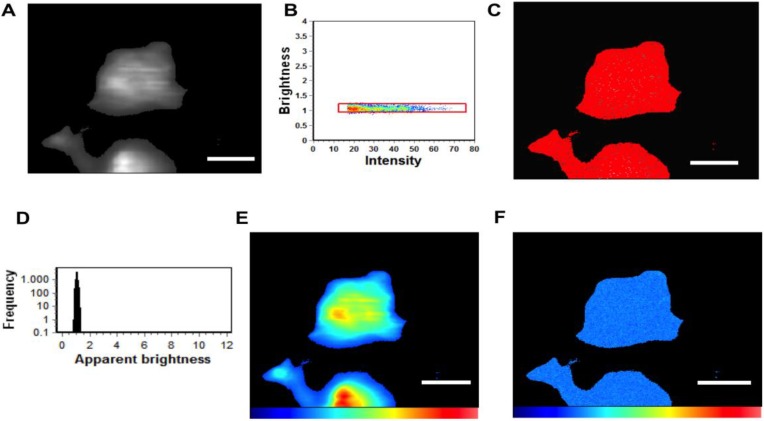
N&B Analysis of monomeric EGFP. (**A**) TIRF microscopy was used to image monomeric EGFP expressed in HEK293 cells; (**B**) Brightness *versus* intensity plot displaying monomers (red box); (**C**) Brightness distribution of EGFP near the PM displaying monomers (red) highlighted in B; (**D**) Frequency *versus* apparent brightness plot demonstrates a tightly correlated monomeric EGFP; (**E**) TIRF intensity image displaying monomeric EGFP distribution near the PM (blue = least intense; red = most intense); (**F**) Cellular brightness map of monomeric EGFP from the TIRF image (blue = least intense; red = most intense). Scale bar = 18 µm.

**Figure 5:**
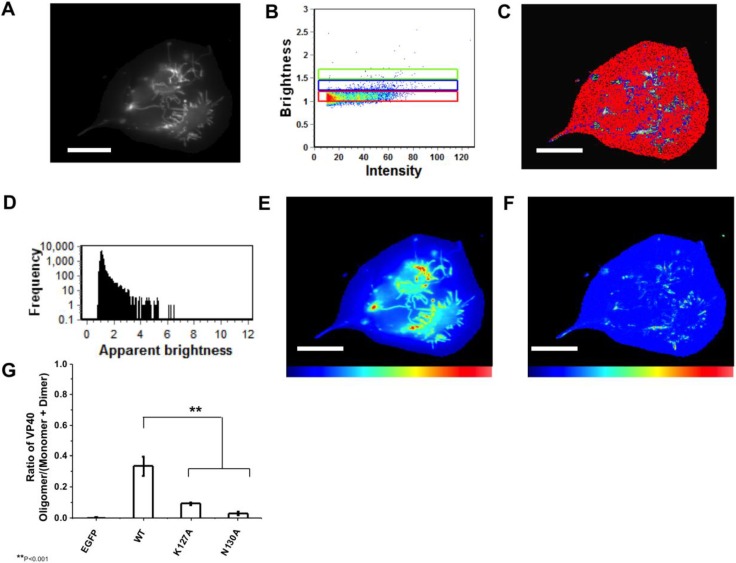
N&B Analysis of monomeric WT VP40. (**A**) TIRF microscopy was used to image EGFP-VP40 expressed in HEK293 cells elucidating extensive VP40 enriched PM protrusions; (**B**) Brightness *versus* intensity plot displaying monomers and dimers (red box), trimers and tetramers (blue box), and hexamers to octamers (green box); (**C**) Brightness distribution of EGFP at or on the PM displaying monomers and dimers (red), trimers and tetramers (blue), and hexamers to octamers (green) highlighted in B; (**D**) Frequency *versus* apparent brightness plot of EGFP-VP40 demonstrates significant oligomerization of VP40 at or near the PM of HEK293 cells; (**E**) TIRF intensity image displaying EGFP-VP40 distribution at or near the PM (blue = least intense; red = most intense). VP40 oligomers are enriched in the cellular protrusions; (**F**) Cellular brightness map of EGFP-VP40 from the TIRF image demonstrates the exclusive localization of VP40 oligomers to membrane protrusion sites (blue = least intense; red = most intense); (**G**) Histogram plot of the ratio of VP40 oligomers/VP40 (monomers+dimers) to demonstrate the reduction of PM oligomerization of K127A and N130A is statistically significant. One-way ANOVA analysis was used to calculate the standard error of the mean and *p*-value. ** *p* < 0.001. Scale bar = 18 µm.

**Figure 6:**
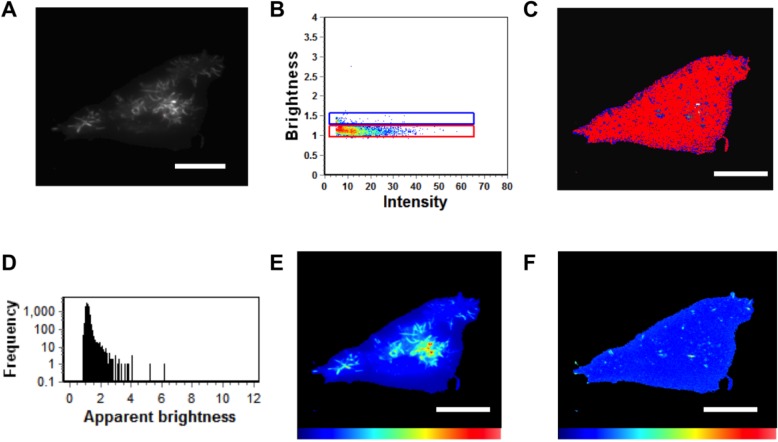
N&B Analysis of K127A. (**A**) TIRF microscopy was used to image EGFP-VP40-K127A expressed in HEK293 cells elucidating VP40 enriched PM protrusions. (**B**) Brightness *versus* intensity plot displaying monomers and dimers (red box) and trimers and tetramers (blue box). Note that little to no hexamers are detected in the brightness *versus* intensity plot. (**C**) Brightness distribution of K127A at or on the PM displaying monomers or dimers (red) and trimers and tetramers (blue). (**D**) Frequency *versus* apparent brightness plot demonstrates significantly lower oligomerization of K127A (See Figure 5G also) at or near the PM of HEK293 cells. (**E**) TIRF intensity image displaying EGFP-VP40-K127A distribution at or near the PM (blue = least intense; red = most intense). VP40 oligomers are enriched in the cellular protrusions. (**F**) The cellular brightness map of EGFP-VP40-K127A from the TIRF image demonstrates the significant reduction in oligomerization compared to WT (blue = least intense; red = most intense). Scale bar = 18 µm.

**Figure 7:**
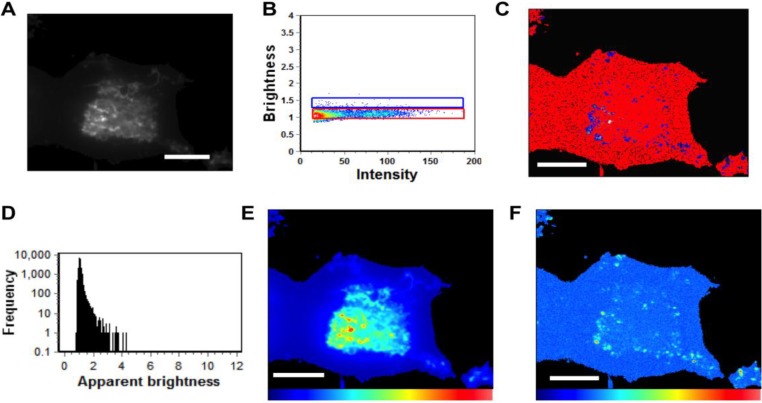
N&B Analysis of N130A. (**A**) TIRF microscopy was used to image EGFP-VP40-N130A expressed in HEK293 cells elucidating a low level of N130A enriched PM protrusions. (**B**) Brightness *versus* intensity plot displaying monomers and dimers (red box) and trimers and tetramers (blue box). Note that little to no hexamers were detected in the brightness *versus* intensity plot. (**C**) Brightness distribution of N130A at or on the PM displaying monomers and dimers (red) or trimers and tetramers (blue). (**D**) Frequency *versus* apparent brightness plot demonstrates significantly lower oligomerization of N130A at or near the PM of HEK293 cells. (**E**) TIRF intensity image displaying EGFP-VP40-N130A distribution at or near the PM (blue = least intense; red = most intense). N130A enrichment at the PM and protrusion sites is greatly reduced compared to WT VP40 and even K127A (See Figure 5G). (**F**) Despite the presence of intense punctae of N130A at the PM, the cellular brightness map of EGFP-VP40-N130A from the TIRF image demonstrates the significant reduction in oligomerization or PM protrusions compared to WT and K127A (blue = least intense; red = most intense) (See Figure 5G). Scale bar = 18 µm.

Monomeric EGFP was first expressed as a control in HEK293 cells to calibrate the instrument for detection of oligomerization (Figure 4) in five independent experiments. Subsequently, cells expressing EGFP-VP40 were imaged in seven independent experiments and as previously reported [14,27], the predominant form of VP40 on the PM were monomers and dimers (See Figure 5). Hexamers and larger oligomers (>8) were also enriched but localized almost exclusively to membrane regions extending from the PM (Figure 5A,E). This along with previous studies [13] implies the dimers build higher order oligomers that are then involved in VLP egress. Previously, the sites of protrusion were lost when VP40 mutants were employed that abolished oligomerization [14,17,27] strongly suggesting the membrane protrusion sites require VP40 membrane association and oligomerization, which is also supported by the recent structural observations of VP40 assembly [13]. In order to test the role of the N-terminal domain loop residues in VP40 oligomerization we used TIRF and N&B to assess the oligomerization of K127A (Figure 6) and N130A (Figure 7) in five independent experiments. Both mutations displayed a very low fluorescent intensity of EGFP on the PM (Figure 6 and Figure 7) in a similar fashion to cellular localization experiments shown in Figure 2. K127A and N130A, which had a similar level of monomers and dimers as WT, also exhibited a drastic loss of brightness or oligomers (variance/intensity) at the PM (see Figure 5B,C,G, Figure 6B,C, and Figure 7B,C) as well as loss of membrane protrusion (VLP egress sites) sites. This suggests a lack of EGFP clustering and oligomerization for these mutations. Indeed, brightness *versus* intensity plots revealed little oligomerization for these mutants (Figure 5B,C, Figure 6B,C and Figure 7B,C) with the predominant species in these cells being dimers and monomers. Analysis of frequency *versus* apparent brightness plots, for WT and mutations oligomer to monomer plus dimer ratios, demonstrated a statistically significant reduction in VP40 oligomerization when membrane association was ablated for K127A or N130A (Figure 5G).

### 3.4. Viral Egress Studies

**Figure 8:**
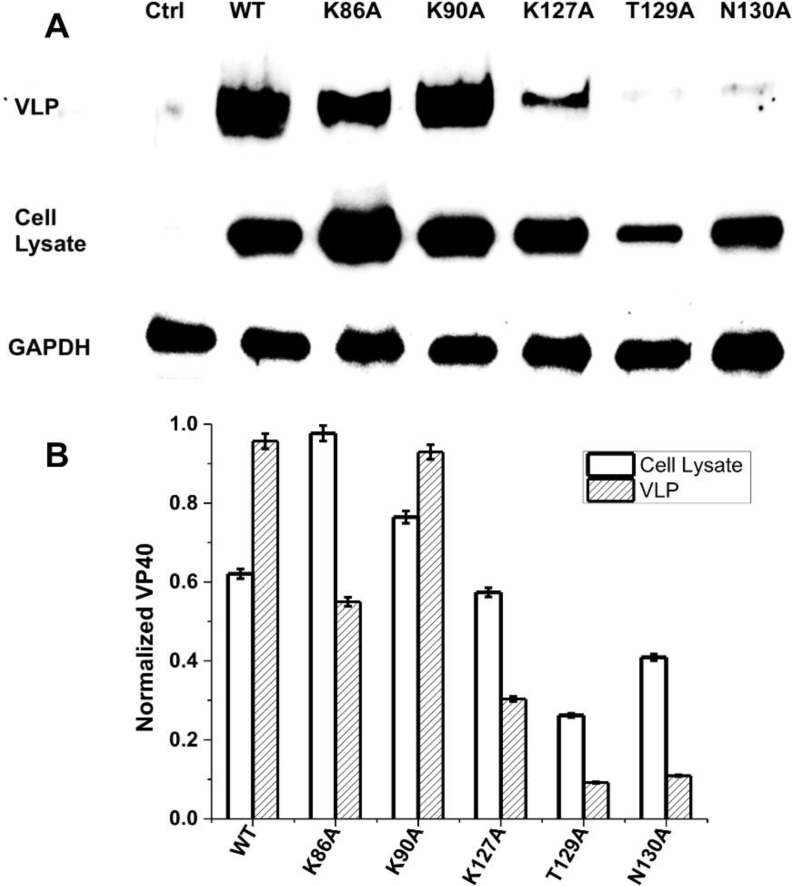
VLP analysis of VP40 and respective mutations. CHOK-1 cells were transfected with plasmid DNA encoding either empty EGFP plasmid (control) or WT VP40, K86A, K90A, K127A, T129A, or N130A EGFP fusion constructs. (**A**) The cell lysate and VLPs were collected after 48 h as described previously [14,17,21] and subjected to Western blot with anti-EGFP. The ratio of cell lysate to VLPs was maintained for each sample with GAPDH used as a loading control for total cellular density. (**B**) Image J was used to quantify band density for each sample repeated in triplicate in order to determine the standard deviation. The band densities were first normalized to the GAPDH loading control and then normalized with respect to VP40 cell lysate band density. The normalized average band intensity is shown for cell lysate VP40 and VLP VP40 for each construct.

To investigate the role of the N-terminal loop in VLP release we collected VLPs for each construct 48 h post-transfection as described in the experimental procedures. The isolated VLPs and cell lysates were subjected to Western blot (Figure 8A) for the EGFP tag using blotting for GAPDH as a loading control. Western blot results demonstrated that mutation of any single residue in the N-terminal loop region harboring Lys^127^, Thr^129^ or Asn^130^ greatly reduced VLP release (Figure 8A). K86A and K90A, however, had similar release of VLPs to WT (Figure 8) in line with PM localization and oligomerization data. The VLP analysis was performed in triplicate and the density of VLP VP40, cytoplasmic VP40, and GAPDH loading control bands were quantified using Image J. These measurements were used to normalize signal to the GAPDH loading control and quantitatively demonstrate the reduction in VLP formation for K127A, T129A, and N130A (Figure 8B). Taken together, the loop region harboring residues Lys^127^, Thr^129^ and Asn^130^ is important in VP40 PM localization, oligomerization and VLP release.

## 4. Discussion

In this study we have identified an important loop region in VP40 that is critical to effective PM localization, VP40 oligomerization, and VLP formation. This region, which resides in the N-terminal domain (See Figure 1A,B), is highly conserved among the five ebolaviruses identified to date. Lys^127^ and Asn^130^ are invariably present among the five ebolaviruses (Bundibugyo virus, BDBV; Ebola virus, EBOV; Reston virus, RESTV; Sudan virus, SUDV; and Tai Forest virus, TAFV) while position 129 is a threonine in EBOV and a serine in the other four ebolaviruses. However, a threonine is also present at position 128 in the BDBV and TAFV. Thr^129^ and Asn^130^ seem to be a bit more critical than Lys^127^ as mutation to alanine more dramatically reduced VLP formation. In contrast, mutation of cationic charge in the N-terminal domain as a whole (K86A and K90A) didn’t lead to nonspecific effects as these mutations localized analogous to WT and displayed robust VLP formation. Stability of loop region mutations doesn’t seem to be an issue as they expressed similar to WT, had overlapping CD spectra, and formation of perinuclear ring structures in cells was not observed which is associated with VP40 octameric ring formation [13].

**Figure 9:**
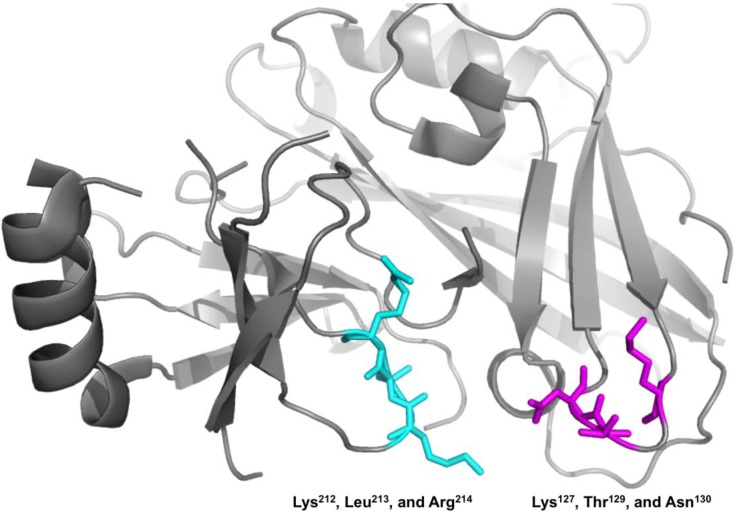
The Lys^127^, Thr^129^, Asn^130^ N-terminal loop region is on the same interface as a C-terminal residue sequence previously identified to be important for VP40 budding. Lys^127^, Thr^129^, Asn^130^ (magenta) in the NTD (PDB ID: 4LDB) are adjacent and on the same interface as a CTD sequence (Lys^212^, Leu^213^, and Arg^214^ in cyan) previously shown to be important to VP40 PM localization and budding.

What exactly does this VP40 loop region interact with? At this juncture the molecular details are unknown but various protein or lipid interactions are possible. Interestingly, the loop region identified here is adjacent to and on the same interface as a CTD sequence (Lys^212^, Leu^213^, and Arg^214^) (See Figure 9) that was previously shown to be important for VP40 budding [28]. Mutation of this KLR sequence resulted in a drastic decrease in VLP release, reduced PM localization, and altered VP40 oligomerization [28]. L213A was the most significant at knocking out budding [28] and TIRF studies indicated previously that L213A had a drastic reduction in PM binding and oligomerization in cells as well as membrane penetration *in vitro* [14]. Previous studies also have speculated that the region near Lys^212^ may form part of a weak interaction of the CTD and NTD, release of which may drive spontaneous oligomerization of VP40 [29]. For instance, C-terminal truncations of VP40 can lead to efficient hexamerization [16]. Reynard *et al.* also found that mutation of proline residues (Pro^205^, Pro^211^, and Pro^215^) in the CTD adjacent to the KLR sequence altered PM localization and budding of VLPs [29]. These mutations actually induced stronger binding to Sec24C and the author’s proposed that these mutations are not as efficient in dissociating from Sec24C, thus limiting their PM localization and egress [29]. In principle then, a similar effect could be proposed for the NTD loop region mutated here that lies adjacent to the C-terminal domain. However, in all cells for which we applied our cellular oligomerization analysis, the ability of VP40 to oligomerize was significantly inhibited suggesting again that defects in transport, membrane binding, protein-protein interactions, or oligomerization to VP40 filaments required for egress [13] are the most likely causes of the decrease in VLP formation. Additionally, we didn’t observe the enriched perinuclear structures of VP40 observed for VP40 mutations that enhanced binding to Sec24C and altered transport [29]. Moreover, this loop is exposed in the VP40 octameric ring so mutations are not expected to inhibit ring formation or RNA binding.

A number of cellular studies have elucidated important regions or residues in VP40 for cellular budding and egress to occur. For example, dimerization of VP40 is mediated by a NTD alpha-helix (Thr^112^ and Leu^117^) while hexamerization occurs through a CTD interface containing Ile^307^ and Met^241^ [13]. VP40 interacts with Sec24C through a hydrophobic patch in the C-terminal domain (residues 303–307), which is required for COPII vesicle transport [24]. The PTAP and PPxY late domains reside at the N-terminus of VP40 and are important for interactions with the ESCRT machinery and likely play a role in enhancing budding [30,33,34]. Pro^283^ and Pro^286^ have been shown to be important to VP40 localization to membrane rafts [25] while a sequence centered around Pro^53^ may regulate an interaction with a novel host factor to achieve budding [35]. Similar to the aforementioned KLR sequence, the NTD sequence 96-LPLGVA-101 in EBOV and 84-LPLGIM-89 in MARV are also required for efficient budding [36].

Membrane binding is thought to occur through electrostatic interactions between the C-terminal domain (Lys^224^, ^225^, ^274^, and ^275^) and the anionic cytoplasmic PM leaflet [13]. Additionally, a hydrophobic loop region in the C-terminal domain (Ile^293^, Val^295^, and Leu^298^) selectively penetrates membranes that recapitulate the PM cytoplasmic leaflet but not the cytoplasmic leaflet of the nuclear membrane [14]. To the best of our knowledge and analysis, the loop region examined here (Lys^127^, Thr^129^, and Asn^130^) is distinct in terms of its role and may do something mechanistically different in egress than the interactions highlighted above. The recent and elegant structural information that has become available on VP40 [13] should allow researchers to better probe and understand VP40-host cell interactions including the loop region identified herein.

The residues in this N-terminal loop aren’t strictly conserved in MARV VP40 but without structural information on MARV VP40 it is difficult to predict what these N-terminal domain loops may look like. This may suggest MARV and EBOV have differential means of lipid-protein or protein-protein interactions. This is in addition to possible differential mechanisms of intracellular trafficking of MARV and EBOV VP40, for which several recent studies have keenly elucidated some of the mechanisms of MARV VP40 intracellular trafficking [37]. Additionally, both EBOV VP40 and MARV VP40 have been shown to be Tyr phosphorylated in cells [38,39] so phosphorylation of Thr^129^ (or Ser in other ebolaviruses) is something we cannot rule out at this time.

While a number of regions of VP40 that are key determinants of trafficking or release have been identified, much less mechanistic information is available on the molecular basis of VP40 interactions with human proteins or membrane lipids. Nonetheless, more detailed efforts are warranted to investigate the role of these amino acids in VP40 mediated assembly including investigation of interactions with other proteins, the PM, or COPII vesicle components. The loop region identified in this study in addition to several previous sequences revealed within the N- and C-terminal domains [28,29,36] should serve as a template to fully understand the transport, oligomerization, and budding of VP40 in human cells.
